# BMI z-score as a prognostic factor for height velocity in children treated with recombinant human growth hormone due to idiopathic growth hormone deficiency

**DOI:** 10.1007/s12020-024-03984-0

**Published:** 2024-08-06

**Authors:** Joanna Budzulak, Katarzyna Anna Majewska, Andrzej Kędzia

**Affiliations:** Department of Pediatric Diabetes, Auxology and Obesity, Poznan University of Medical Sciences, Poznań, Poland

**Keywords:** Children, Short stature, Growth hormone, Body mass index

## Abstract

**Purpose:**

Growth hormone deficiency (GHD) causes growth disturbances during childhood. The most recommended treatment of GHD is the administration of recombinant human growth hormone (rhGH). Recent studies have proved that well-nourished GHD children respond better to rhGH therapy compared to undernourished individuals. The aim of this study was to analyze nutritional status along with height velocity in GHD children during the first two years of rhGH therapy, and to estimate the optimal BMI z-score range in which these children achieve the best growth results.

**Methods:**

This retrospective analysis included 80 prepubertal idiopathic GHD children treated with rhGH. Anthropometric data were obtained from medical records made at an initial visit and then follow-up visits after 12 and 24 months of treatment. The body mass index (BMI) was calculated and standardized into z-score, basing on Cole’s LMS method. Then, the BMI z-score was analyzed in relation to the parameters of growth response.

**Results:**

The higher the BMI z-score at treatment entry, the greater the increase in height during the first twelve months of rhGH therapy. BMI z-score ≥0 noted at the beginning of each year of the treatment are associated with significantly better growth increments throughout the first and the second years of the therapy.

**Conclusion:**

Prepubertal idiopathic GHD children with BMI z-score below 0 would probably benefit from the improvement of their nutritional status prior to the rhGH treatment beginning. It seems that increasing BMI z-score to obtain values between 0 and 1 would be optimal for the growth process.

## Introduction

Short stature is usually defined as height below 2 standard deviations (-2SD) or the 3rd percentile compared to individuals of the same age and gender. The majority of children with short stature present normal variants of growth, including constitutional delay in growth and puberty as well as familial short stature [1, 2]. However, growth disturbances might also appear due to pathological health conditions, such as growth hormone deficiency (GHD), characterized by impaired growth hormone (GH) secretion [3].

Growth hormone is a 191 amino acid single-chain polypeptide produced and released by the pituitary gland in a pulsatile manner. It stimulates body growth affecting tissues both directly, and indirectly through the insulin-like growth factor type-1 (IGF-1) [4, 5]. Both GH and IGF-1 promote bone formation and linear growth through their cognate receptors located on the epiphyseal growth plate [6].

In children with GHD the primary goal of treatment is to normalize growth, which is based on an administration of recombinant human growth hormone (rhGH) in daily injections. The effectiveness of this therapy depends on many factors, including parental height, drug dosage, age at the beginning of treatment, comorbidities, as well as nutritional status [7, 8].

Numerous studies have shown that malnutrition alters the GH/IGF-1 axis and the response of growth plate to GH and IGF-1 [9–11]. It has been also observed that undernourished GHD children achieve worse rhGH therapy results than those with normal body weight [8, 10]. Therefore, it is likely that an assessment of the nutritional status could help to predict GHD treatment outcomes, while its improvement could contribute to better treatment results in terms of height gain.

The most common, simple and non-invasive method of nutritional status assessment is the body mass index (BMI). In the pediatric population it is usually standardized into z-score, as the BMI reference values change with age in children.

The aim of this study was to analyze the nutritional status along with height velocity in GHD children during the first two years of rhGH therapy, and to estimate the optimal BMI z-score range in which these children achieve the best growth results.

## Materials and methods

### Subjects

This retrospective study was based on the medical data analysis of 80 children with idiopathic GHD, treated with rhGH in the period of 2012–2022 at The Karol Jonscher Memorial Clinical Hospital of the Poznan University of Medical Sciences. Data were obtained from medical records made at the first visit, when treatment was started, and then during follow-up visits after 12 and 24 months.

### Inclusion and exclusion criteria

All patients met the diagnostic criteria for GHD and were eligible for the Polish rhGH treatment program. The study included only short-statured patients (height below -2SDS before the start of treatment) diagnosed with isolated GHD without comorbidities. GH secretion was assessed in two stimulation tests (insulin, clonidine or glucagon). In all children obtained peak GH serum levels were lower than 10 ng/ml, but higher than 3 ng/ml, indicating partial GHD. Children with other pituitary hormone deficiencies, multihormonal hypopituitarism, as well as congenital defect syndromes were excluded from the analysis. Children diagnosed with asthma, epilepsy, intrauterine growth restriction (IUGR), born small for gestational age (SGA) and with celiac disease were also excluded. Study participants did not present thyroid dysfunction throughout the observation. All of them were prepubertal during the observation period (Tanner stage 1). Children were treated with rhGH (Genotropin® or Omnitrope®) at the dose range 0.160–0.190 mg/kg/week in daily injections, according to the consensus guidelines [12, 13].

### Auxological and clinical measurements

All participants were evaluated by pediatric endocrinologists during initial visit and then follow-up visits after 12 and 24 months of treatment. Height was assessed to the nearest 0.1 cm using the same stadiometer. The children were measured standing in an upright position with the head positioned to the Frankfurt plane. Body weight was determined with an accuracy of 0.05 kg. Height SDS (hSDS) was calculated according to the local centile charts for the child’s age and sex [14], by the following formula:

$${\mathrm{hSDS}} = \frac{{\left( {{\mathrm{actual}}\,{\mathrm{height}} - {\mathrm{height}}\,{\mathrm{at}}\,50{\mathrm{th}}\,{\mathrm{centile}}} \right)}}{{\left[ {0.5 \times \left( {{\mathrm{height}}\,{\mathrm{at}}\,50{\mathrm{th}}\,{\mathrm{centile}} - {\mathrm{height}}\,{\mathrm{at}}\,3{\mathrm{th}}\,{\mathrm{centile}}} \right)} \right]}}$$

The effectiveness of the treatment was assessed by the estimation of height velocity (HV) and the increase in hSDS during each year of therapy. Height velocity (HV) was calculated as a difference in height between consecutive annual follow-up visits. The body mass index (BMI) was standardized for sex and age and expressed as a BMI z-score, based on the Cole’s LMS method [15]. The LMS parameters were derived from Polish growth references for pre-school (2012) and school-aged children (2010) [16, 17]. For the purpose of the statistical analysis, participants were divided into sub-groups, according to their BMI z-score values recorded at the treatment beginning. Due to the fact that 75% of the studied children had initial BMI z-score below 0 and only 25% of them equal or above 0, additionally three sub-groups were distinguished depending on BMI z-score value: <−1, in the range [−1;0) and ≥0.

$${\mathrm{BMI}} = \frac{{{\mathrm{weight}}\,{\mathrm{in}}\,{\mathrm{kilograms}}}}{{{\mathrm{height}}\,{\mathrm{in}}\,{\mathrm{metres}}^2}}$$

$${\mathrm{BMI}}\,{\mathrm{z}} - {\mathrm{score}} = \frac{{\left( {\frac{{{\mathrm{BMI}}}}{{\mathrm{M}}}} \right)^L - 1}}{{{\mathrm{L}} \times {\mathrm{S}}}}$$

### Statistical analysis

Statistical analysis was performed using an IBM SPSS Statistics 26. The value of p < 0.05 was considered as statistically significant. The Shapiro-Wilk test was performed to test the normality assumption. In order to compare quantitative variables in two groups, the Mann−Whitney U test was applied, while in the case of more than 2 groups, the Kruskal-Wallis test was performed, with the Bonferroni correction as a post-hoc procedure. To investigate the differences between the periods of observation, non-parametric tests were used: the Wilcoxon signed-rank test for two groups and the Friedman two-way analysis for more than two groups. Due to the lack of a normal distribution of variables, the Spearman correlation test was made to investigate mutual relations.

## Results

A total of 80 GHD pediatric patients were enrolled for this study: 55 boys (69%) and 25 girls (31%), with the mean chronological age 7.88 ± 1.76. Detailed group characteristics are presented in Table 1. At the initial visit the girls had significantly higher mean hSDS compared to the boys (p = 0.023). There was no significant difference in BMI z-score values between genders at initial visit (p = 0.783), 12-month (p = 0.652) and 24-month (p = 0.996) follow-up visits. According to the whole study group, the mean height as well as hSDS improved significantly after both 12 and 24 months of treatment (p < 0.001). The subjects’ average body weight also increased significantly throughout the two years of therapy (p < 0.001). However, changes in the mean BMI z-score did not reach statistical significance (p = 0.363).

**Table 1:** Characteristics of the study group at all follow-up visits

Parameter	Initial visit	12-month follow-up visit	24-month follow-up visit
Total number of subjects	n = 80		
Boys/Girls	n = 55 (69%)/ n = 25 (31%)		
Age in years (mean ± SD)	7.88 ± 1.76	8.90 ± 1.77	9.91 ± 1.77
Boys/Girls	7.76 ± 1.78/8.16 ± 1.73	8.78 ± 1.79/9.18 ± 1.73	9.79 ± 1.79/10.17 ± 1.73
Height in cm (mean ± SD)	114.41 ± 9.30	123.28 ± 9.45	130.34 ± 9.53
Boys/Girls	113.82 ± 9.13/115.72 ± 9.72	122.59 ± 9.12/124.78 ± 10.17	129.53 ± 9.12/132.12 ± 10.32
hSDS (mean ± SD)	−2.89 ± 0.71	−2.08 ± 0.71	−1.71 ± 0.74
Boys/Girls	−3 ± 0.76/−2.64 ± 0.55	−2.14 ± 0.75/−1.96 ± 0.62	−1.76 ± 0.79/−1.61 ± 0.62
Weight in kg (mean ± SD)	20.47 ± 4.95	24.18 ± 6.08	27.85 ± 7.50
Boys/Girls	19.89 ± 3.96/21.77 ± 6.55	23.35 ± 4.66/26 ± 8.22	26.86 ± 5.39/30.05 ± 10.61
BMI z-score (mean ± SD)	−0.53 ± 0.93	−0.62 ± 0.99	−0.60 ± 0.98
Boys/Girls	−0.56 ± 0.77/−0.46 ± 1.23	−0.68 ± 0.86/−0.49 ± 1.25	−0.64 ± 0.81/−0.52 ± 1.30

*hSDS* height standard deviation score, *BMI* body mass index

Parameters of height velocity (HV), as well as changes in hSDS (ΔhSDS) obtained during the first year of the treatment were significantly higher than in the following period (p < 0.001) [Table 2]. In the first year of rhGH therapy there were no statistically significant gender differences in HV (p = 0.618), while the average ΔhSDS was found to be significantly better among boys (p = 0.035). For comparison, in the second year of the treatment the mean HV (p = 0.266) as well as ΔhSDS (p = 0.471) did not reach a statistical significance between genders regarding the assessed parameter. There was no significant difference in the average weight gain (p = 0.922) and the annual BMI z-score (p = 0.220) between the first and the second year of therapy.

**Table 2:** Changes in antropomethric measurements throughout two years of rhGH therapy

Parameter	First year of treatment	Second year of treatment
HV (mean ± SD)	8.86 ± 1.27	7.06 ± 1.12
Boys/Girls	8.78 ± 1.25 / 9.06 ± 1.33	6.93 ± 1.02 / 7.35 ± 1.29
ΔhSDS (mean ± SD)	0.81 ± 0.35	0.37 ± 0.23
Boys/Girls	0.86 ± 0.35 / 0.68 ± 0.31	0.38 ± 0.23 / 0.35 ± 0.23
Weight gain (mean ± SD)	3.70 ± 1.70	3.68 ± 2.08
Boys/Girls	3.46 ± 1.32 / 4.23 ± 2.28	3.51 ± 1.35 / 4.05 ± 3.16
Annual BMI z-score^a^ (mean ± SD)	− 0.57 ± 0.93	− 0.61 ± 0.97
Boys/Girls	− 0.62 ± 0.77 / −0.47 ± 1.22	− 0.66 ± 0.82 / −0.50 ± 1.26

*HV* height velocity, *hSDS* height standard deviation score, *BMI* body mass index

^a^The annual BMI z-score was calculated as a mean value of the BMI z-score from two visists: at the beginning and at the end of each year of treatment

Spearman’s rank correlation was computed to assess the relationship between the BMI z-score values and the parameters of growth response [Table 3]. The results indicate that there was a significant positive correlation between the mean BMI z-score at the initial visit and HV in the first year of treatment. The higher the BMI z-score at treatment entry, the greater the increase in height during the first twelve months of therapy. The same significant positive correlation was observed between average BMI z-score values at 12-month and 24-month follow-up visits and HV in the first year of treatment. However, no significant relationship was found between the mean BMI z-score at all follow-up visits and ΔhSDS in this period. According to the second year of therapy, there was no significant correlation between the BMI z-score at all follow-up visits and HV as well as ΔhSDS.

**Table 3:** Spearman’s rank correlation coefficient values for correlations between the BMI z-score at follow-up visits and ΔhSDS and HV during the first two years of rhGH therapy

BMI z-score	Statistical values	ΔhSDS in the 1st year of treatment	ΔhSDS in the 2nd year of treatment	HV in the 1st year of treatment	HV in the 2nd year of treatment
Initial visit	r	0.105	0.048	0.276*	0.067
	p	0.353	0.671	0.013	0.553
12-month follow-up visit	r	0.054	0.078	0.228*	0.151
	p	0.633	0.490	0.041	0.181
24-month follow-up visit	r	0.100	0.120	0.246*	0.170
	p	0.379	0.290	0.028	0.132

*HV* height velocity, *hSDS* height standard deviation score, *BMI* body mass index, *for p < 0.05

The study group was also divided into three sub-groups depending on the BMI z-score values recorded at the initial visit. These were then analyzed in relation to HV [Table 4]. Prior to the treatment 75% of patients appeared to have a BMI z-score below 0. At the initial visit 25% of children presented a BMI z-score below −1 (<−1), while 50% of children had a BMI z-score in the range [−1;0) and 25% had a BMI z-score equal to or above 0 ( ≥ 0). The obtained results indicated a significant difference in HV in the first year of rhGH treatment among BMI z-score sub-groups (p = 0.032). The mean HV in children with BMI z-score ≥0 at the initial visit was significantly higher than in the groups with a BMI z-score <−1 and [−1;0). There was no significant difference in HV between groups with a BMI z-score <−1 and [−1;0) at the initial visit in this period of treatment. Furthermore, the results of this analysis show that there was also a significant difference in HV between the sub-groups on 12-month (p = 0.036) and 24-month (p = 0.027) follow-up visit. Again, the mean HV in the second year of therapy for the group with a BMI z-score ≥0 was significantly higher than in subjects with a BMI z-score <−1 and [−1;0). There was no significant difference in HV during the second year of therapy between groups with a BMI z-score <−1 and [−1;0) measured at the 12-month and 24-month follow-up visits.

**Table 4:** The association between BMI z-score ranges and HV throughout the first two years of rhGH therapy

HV	Type of visit	BMI z-score ranges	*N*	Mean HV ± SD	p value
The 1st year of treatment	Initial visit	<−1.00	20	8.58 ± 0.81	0.032*
		[−1.00;0.00)	40	8.68 ± 1.28	
		≥0.00	20	9.52 ± 1.46	
	12-month follow-up visit	<−1.00	28	8.69 ± 0.93	0.061
		[−1.00;0.00)	32	8.63 ± 1.35	
		≥0.00	20	9.50 ± 1.41	
	24-month follow-up visit	<−1.00	28	8.73 ± 1.02	0.057
		[−1.00;0.00)	32	8.64 ± 1.43	
		≥0.00	20	9.42 ± 1.22	
The 2nd year of treatment	Initial visit	<−1.00	20	7.09 ± 0.97	0.361
		[−1.00;0.00)	40	6.89 ± 1.11	
		≥0.00	20	7.37 ± 1.26	
	12-month follow-up visit	<−1.00	28	6.88 ± 0.93	0.036*
		[−1.00;0.00)	32	6.87 ± 0.94	
		≥0.00	20	7.63 ± 1.44	
	24-month follow-up visit	<−1.00	28	6.91 ± 0.96	0.027*
		[−1.00;0.00)	32	6.78 ± 0.89	
		≥0.00	20	7.72 ± 1.41	

*HV* height velocity, *BMI* body mass index, *p < 0.05

## Discussion

Even though rhGH therapy in GHD children is generally considered to be safe and effective [18], some statistics indicate that the rate of poor responders may reach up to 30% [19]. There are several known factors which can influence the treatment results, including the severity of growth hormone deficiency, age, birth weight and bone age delay [20, 21]. It has been also suggested that malnutrition can impair treatment results [22]. It seems that well-nourished GHD children should respond better to rhGH therapy than those with a poor nutritional status [8, 10, 23, 24].

Height gain during the first year of treatment with rhGH is thought to be an important predictor of the final treatment outcome [21, 25]. It is considered that in prepubertal idiopathic GHD children the increase of 0.5 in hSDS value (ΔhSDS) over this period allows the attainment of a final height increment of 1 SDS [21]. In our study, the average ΔhSDS over the first twelve months of therapy reached 0.81 and was noticeably better among boys. In the second year of treatment it was less pronounced.

These results stay in line with observations that at the beginning of rhGH therapy there is usually a rapid and significant growth increment, commonly called “catch-up growth”, which gradually decreases over time [7, 26, 27]. Our study also presented significantly higher ΔhSDS among boys in the first year of therapy. However, most of the scientific data does not indicate sex-related differences in rhGH treatment outcomes [28–30]. The above observation was probably the result of more pronounced height deficiency in boys of our study group.

BMI standardized into z-score allows the assessment of the nutritional state in children according to their age and gender. In our research, no significant changes in mean BMI z-score values were found between the initial visit, after 12 months and after 24 months of treatment, which suggest that this parameter remains relatively stable during the first years of the therapy. In numerous studies it has been observed that patients with untreated GHD present specific alterations in body composition - increased fat mass, decreased lean and bone mass, which all improve after following rhGH therapy [31]. However, BMI is not an indicator of body composition, because subjects with similar BMI values might present different ratio of fat mass to fat-free mass [32]. Therefore, changes in body composition resulting from rhGH treatment, might not lead to changes in BMI values.

In our study we investigated the impact of the BMI z-score on height velocity during the first and the second year of rhGH therapy. The results of this analysis indicate that the higher the BMI z-score noted at the initial visit, the greater the height velocity during the first twelve months of the therapy. This positive relation was also observed in reference to BMI z-score values measured at consecutive follow-up visits: after 12 months and after 24 months of treatment, probably because the average BMI z-score had not changed meaningfully over time.

It is worth to mention that there is large amount of evidence that nutritional status impacts the growth process affecting the GH/IGF1 axis [11, 33, 34]. Patients with undernutrition usually present decreased free IGF-1 levels and develop GH resistance. Diet deficiency in energy and protein might lower IGF-1 values even during short-term and mild restrictions. Moreover, it has been shown, that insufficient food intake in children with GHD leads to significant decrease in IGF-1 levels despite ongoing rhGH therapy [10], which implies that poor nutrition might alter rhGH treatment results.

On the other hand, excessive body weight might also disturb the GH/IGF-1 axis [35]. A systematic review and meta-analysis from the year 2021, involving 58 studies, has shown a significant negative relationship between BMI-SDS and peak stimulated GH concentrations [36]. However, despite lower GH levels, obese patients present enhanced GH sensitivity [10], while IGF-1 levels are reported as decreased, normal or elevated [35]. Obesity is commonly associated with accelerated growth [11, 37], although excessive body weight might affect growth differently, depending on the pubertal stage.

A study of Kempf et al show, that in early childhood obese children show increased growth, together with elevated IGF-1, insulin and leptin levels. In contrast, obesity in puberty was linked to a reduction in growth velocity, a decrease in levels of IGF-1, as well as testosterone in boys and estradiol in girls [38]. Another study conducted among 460 prepubertal children with idiopathic, organic, complete and partial GHD has shown that among all types of GHD, children with obesity had significantly better rhGH treatment outcomes, compared to non-obese participants [35].

Given the above, it seems that increasing body weight in prepubertal GHD children with lower BMI values, might help to achieve improved growth results. The catch-up growth observed in the first months after the beginning of rhGH therapy requires a large amount of calories [39]. Therefore, we presume that children with higher BMI values would be more likely to meet energy requirements needed for an effective catch-up growth.

In our study we aimed to estimate the ranges of BMI z-score which would be optimal for the best rhGH treatment outcome. The obtained results show that BMI z-score ≥0 noted at the beginning of each year of the therapy lead to better height gains throughout the first and the second years of the therapy. However, according to recommendations of the World Health Organization (WHO) regarding BMI z-score cut-off points in nutritional state assessment during childhood, values between 1 and 1.99 are classified as overweight, and above 2 as obesity [40]. It must be emphasized that excessive body weight is a risk factor for metabolic disorders, including diabetes type 2 and cardiovascular diseases [41]. It is assumed that GHD patients are more prone to develop cardiovascular diseases in the future [42], while rhGH treatment possibly enhances the risk of developing diabetes type 2 [43]. Therefore, BMI z-score ≥1 should not be recommended for GHD children, despite its potential positive effect on growth increments during rhGH treatment.

This study provides results which might be useful in practice for clinicians working with GHD pediatric patients, especially when treated with rhGH. Achieving proper BMI-for-age might help to achieve the best treatment results. However, this analysis has some limitations. We included only prepubertal children in order to avoid the additional influence of sex hormones on growth, therefore the obtained results should not be generalized into the pubertal age. An enlargement of the study group, including patients in various puberty stages could improve an understanding of nutritional state influence on growth during puberty, but the interpretation of those results and distinguishing nutritional aspects from sex hormones action would be a challenge. Another limitation is that our study considers only the BMI z-score as an indicator of nutritional status and, as it was previously mentioned, BMI does not reflect body composition. However, we aimed to find an easy-obtainable and comparable prognostic factor for rhGH treatment outcome and the chosen parameter seems to meet these requirements.

## Conclusion

Nutritional status has proved to be an important factor affecting rhGH treatment outcome. In prepubertal idiopathic GHD children, BMI z-score values ≥ 0 at the beginning of the therapy are associated with better growth results throughout the first two years of its duration. Therefore, nutritional assessment should be an integral part of the care for GHD children. As BMI z-score values remain rather stable in the first years of the therapy, patients with its values below 0 would probably benefit most from nutritional intervention, if it was implemented prior to the beginning of the treatment. It seems that increasing BMI z-score to obtain values in the range between 0 and 1 would be optimal for the growth process during rhGH therapy.

## Data Availability

The data that support the findings of this study are available from the corresponding author upon reasonable request.

## References

[1] D. Rani, R. Shrestha, T. Kanchan, K. Krishan, Short Stature. StatPearls [Internet]. Treasure Island (FL): StatPearls Publishing (2024). https://www.ncbi.nlm.nih.gov/books/NBK556031/. Accessed 23 Jan 2024 32310491

[2] K.A. Majewska, A. Kędzia, P. Kontowicz, M. Prauzińska, J. Szydłowski, M. Świtoński, J. Nowacka-Woszuk, Polymorphism of the growth hormone gene GH1 in Polish children and adolescents with short stature. Endocrine **69**(1), 157–164 (2020). 10.1007/s12020-020-02305-5 32338337 10.1007/s12020-020-02305-5 PMC7343724

[3] J.G. Halas, A. Grimberg, Dilemmas of growth hormone treatment for GH deficiency and idiopathic short stature: defining, distinguishing, and deciding. Minerva Pediatr. **72**(3), 206–225 (2020). 10.23736/S0026-4946.20.05821-1 32274914 10.23736/S0026-4946.20.05821-1 PMC7490116

[4] N. Torlińska-Walkowiak, K.A. Majewska, A. Kędzia, J. Opydo-Szymaczek, Clinical implications of growth hormone deficiency for oral health in children: a systematic review. J. Clin. Med. **10**(16), 3733 (2021). 10.3390/jcm10163733 34442031 10.3390/jcm10163733 PMC8396810

[5] W.F. Blum, A. Alherbish, A. Alsagheir, A. El Awwa, W. Kaplan, E. Koledova, M.O. Savage, The growth hormone-insulin-like growth factor-I axis in the diagnosis and treatment of growth disorders. Endocr. Connect **7**(6), R212–R222 (2018). 10.1530/EC-18-0099 29724795 10.1530/EC-18-0099 PMC5987361

[6] M. Dixit, S.B. Poudel, S. Yakar, Effects of GH/IGF axis on bone and cartilage. Mol. Cell Endocrinol. **519**, 111052 (2021). 10.1016/j.mce.2020.111052 33068640 10.1016/j.mce.2020.111052 PMC7736189

[7] P. Gou, X. Cheng, J. Leng, N. Su, A real-world study of recombinant human growth hormone in the treatment of idiopathic short stature and growth hormone deficiency. Ther. Clin. Risk Manag. **18**, 113–124 (2022). 10.2147/tcrm.s363564 35342293 10.2147/TCRM.S363564 PMC8943615

[8] P.A. Lee, J. Germak, R. Gut, N. Khutoryansky, J. Ross, Identification of factors associated with good response to growth hormone therapy in children with short stature: results from the ANSWER Program®. Int. J. Pediatr. Endocrinol. **2011**(1), 6 (2011). 10.1186/1687-9856-2011-6 21899782 10.1186/1687-9856-2011-6 PMC3168402

[9] M. Caputo, S. Pigni, E. Agosti, T. Daffara, A. Ferrero, N. Filigheddu, F. Prodam, Regulation of GH and GH signaling by nutrients. Cells **10**(6), 1376 (2021). 10.3390/cells10061376 34199514 10.3390/cells10061376 PMC8227158

[10] C.P. Hawkes, A. Grimberg, Insulin-like growth Factor-I is a marker for the nutritional state. Pediatr. Endocrinol. Rev. **13**(2), 499–511 (2015) 26841638 PMC5576178

[11] E. Inzaghi, V. Pampanini, A. Deodati, S. Cianfarani, The effects of nutrition on linear growth. Nutrients **14**(9), 1752 (2022). 10.3390/nu14091752 35565716 10.3390/nu14091752 PMC9100533

[12] Growth Hormone Research S., Consensus guidelines for the diagnosis and treatment of growth hormone (GH) deficiency in childhood and adolescence: summary statement of the GH Research Society. GH Research Society. J. Clin. Endocrinol. Metab. **85**(11), 3990–3993 (2000). 10.1210/jcem.85.11.6984 11095419 10.1210/jcem.85.11.6984

[13] P.F. Collett-Solberg, G. Ambler, P.F. Backeljauw, M. Bidlingmaier, B.M.K. Biller, M.C.S. Boguszewski, P.T. Cheung, C.S.Y. Choong, L.E. Cohen, P. Cohen, A. Dauber, C.L. Deal, C. Gong, Y. Hasegawa, A.R. Hoffman, P.L. Hofman, R. Horikawa, A.A.L. Jorge, A. Juul, P. Kamenický, V. Khadilkar, J.J. Kopchick, B. Kriström, M.L.A. Lopes, X. Luo, B.S. Miller, M. Misra, I. Netchine, S. Radovick, M.B. Ranke, A.D. Rogol, R.G. Rosenfeld, P. Saenger, J.M. Wit, J. Woelfle, Diagnosis, genetics, and therapy of short stature in children: a growth hormone research society international perspective. Horm. Res. Paediatr. **92**(1), 1–14 (2019). 10.1159/000502231 31514194 10.1159/000502231 PMC6979443

[14] I. Palczewska, Z. Niedźwiedzka, Somatic development indices in children and youth of Warsaw. Med. Wieku. Rozwoj **5**(Suppl1), 18–118 (2001) 11675534

[15] T.J. Cole, The LMS method for constructing normalized growth standards. Eur. J. Clin. Nutr. **44**(1), 45–60 (1990) 2354692

[16] Z. Kułaga, A. Grajda, B. Gurzkowska, M. Góźdź, M. Wojtyło, A. Swiąder, A. Różdżyńska-Świątkowska, M. Litwin, Polish 2012 growth references for preschool children. Eur. J. Pediatr. **172**(6), 753–761 (2013). 10.1007/s00431-013-1954-2 23371392 10.1007/s00431-013-1954-2 PMC3663205

[17] Z. Kułaga, M. Litwin, M. Tkaczyk, I. Palczewska, M. Zajączkowska, D. Zwolińska, T. Krynicki, A. Wasilewska, A. Moczulska, A. Morawiec-Knysak, K. Barwicka, A. Grajda, B. Gurzkowska, E. Napieralska, H. Pan, Polish 2010 growth references for school-aged children and adolescents. Eur. J. Pediatr. **170**(5), 599–609 (2011). 10.1007/s00431-010-1329-x 20972688 10.1007/s00431-010-1329-x PMC3078309

[18] M. Maghnie, M.B. Ranke, M.E. Geffner, E. Vlachopapadopoulou, L. Ibáñez, M. Carlsson, W. Cutfield, R. Rooman, R. Gomez, M.P. Wajnrajch, A. Linglart, R. Stawerska, P.E. Clayton, F. Darendeliler, A.C.S. Hokken-Koelega, R. Horikawa, T. Tanaka, H.G. Dörr, K. Albertsson-Wikland, M. Polak, A. Grimberg, Safety and efficacy of pediatric growth hormone therapy: results from the full KIGS Cohort. J. Clin. Endocrinol. Metab. **107**(12), 3287–3301 (2022). 10.1210/clinem/dgac517 36102184 10.1210/clinem/dgac517 PMC9693805

[19] S. Straetemans, J. De Schepper, M. Thomas, S. Tenoutasse, V. Beauloye, R. Rooman, Criteria for first-year growth response to growth hormone treatment in prepubertal children with growth hormone deficiency: do they predict poor adult height outcome? Front. Endocrinol. **10**, 792 (2019). 10.3389/fendo.2019.00792 10.3389/fendo.2019.00792 PMC6888254 31849835

[20] P. van Dommelen, E. Koledova, J.M. Wit, Effect of adherence to growth hormone treatment on 0−2 year catch-up growth in children with growth hormone deficiency. PloS ONE **13**(10), e0206009 (2018). 10.1371/journal.pone.0206009 30356273 10.1371/journal.pone.0206009 PMC6200242

[21] M.O. Savage, P. Bang, The variability of responses to growth hormone therapy in children with short stature. Indian J. Endocrinol. Metab. **16**(Suppl 2), S178–S184 (2012). 10.4103/2230-8210.104034 23565373 10.4103/2230-8210.104034 PMC3603021

[22] World Health Organization (WHO): Malnutrition. https://www.who.int/health-topics/malnutrition (2024). Accessed 23 January 2024

[23] G. Gat-Yablonski, M. Phillip, Nutritionally-induced catch-up growth. Nutrients **7**(1), 517–551 (2015). 10.3390/nu7010517 25594438 10.3390/nu7010517 PMC4303852

[24] A. Majcher, A. Czerwonogrodzka-Senczyna, E. Woźniak, B. Pyrżak, Effect of nutritional status on growth velocity in the first year of growth hormone treatment of children with Growth hormone deficiency. Postępy Nauk Medycznych 27 (2014)

[25] S. Straetemans, M. Thomas, M. Craen, R. Rooman, J. De Schepper, BESPEED: poor growth response during the first year of growth hormone treatment in short prepubertal children with growth hormone deficiency and born small for gestational age: a comparison of different criteria. Int. J. Pediatr. Endocrinol. **2018**, 9 (2018). 10.1186/s13633-018-0064-3 30377433 10.1186/s13633-018-0064-3 PMC6196419

[26] J.M. Wit, B. Boersma, Catch-up growth: definition, mechanisms, and models. J. Pediatr. Endocrinol. Metab. **15**(Suppl 5), 1229–1241 (2002) 12510974

[27] A. Kędzia, K. Majewska, M. Korcz, Do the intervals in growth hormone therapy positively affect the growth velocity? Pediatr. Endocrinol. Diabetes Metab. **26**(3), 113–117 (2020). 10.5114/pedm.2020.97463 32901467 10.5114/pedm.2020.97463

[28] K.R. Essa, A.H. Al-Jumaili, W.A. Hasan, The efficacy of growth hormone therapy on children with growth hormone deficiency treated with recombinant human growth hormone. Med. J. Tikrit Univ. **16**(1), 184–191 (2010)

[29] A. Zubkiewicz-Kucharska, W. Lasota, U. Tumilewicz, A. Matula, The influence of selected factors on the effectiveness of rhGH replacement therapy in children with growth hormone deficiency. Pediatr. Endocrinol. **13**(4), 19–26 (2014). 10.18544/EP-01.13.04.1499

[30] J. Smyczyńska, R. Stawerska, A. Lewiński, M. Hilczer, Auxological and hormonal prognostic factors of growth hormone (GH) therapy effectiveness in children with GH deficiency, available before treatment. Pediatr. Endocrinol. **12**, 9–20 (2013). 10.18544/EP-01.12.02.1445

[31] A. Grimberg, S.A. DiVall, C. Polychronakos, D.B. Allen, L.E. Cohen, J.B. Quintos, W.C. Rossi, C. Feudtner, M.H. Murad, Drug and therapeutics committee and ethics committee of the pediatric endocrine society: guidelines for growth hormone and insulin-like growth Factor-I treatment in children and adolescents: growth hormone deficiency, idiopathic short stature, and primary insulin-like growth Factor-I deficiency. Horm. Res. Paediatr. **86**(6), 361–397 (2016). 10.1159/000452150 27884013 10.1159/000452150

[32] M.R. Licenziati, G. Ballarin, G. Iannuzzo, M.S. Lonardo, O. Di Vincenzo, A. Iannuzzi, G. Valerio, A height-weight formula to measure body fat in childhood obesity. Ital. J. Pediatr. **48**, 106 (2022). 10.1186/s13052-022-01285-8 35729585 10.1186/s13052-022-01285-8 PMC9210685

[33] S. Liang, J. Xue, G. Li, Effects of recombinant human growth hormone administration on cardiovascular risk factors in obese children with relative growth hormone deficiency. Lipids Health Dis. **17**(1), 66 (2018). 10.1186/s12944-018-0721-9 29615058 10.1186/s12944-018-0721-9 PMC5883519

[34] J. Budzulak, K.A. Majewska, A. Kędzia, Malnutrition as the cause of growth retardation among children in developed countries. Ann. Agric. Environ. Med. **29**(3), 336–341 (2022). 10.26444/aaem/148010

[35] A. Yang, S.Y. Cho, M.J. Kwak, S.J. Kim, S.W. Park, D.K. Jin, J.E. Lee, Impact of BMI on peak growth hormone responses to provocative tests and therapeutic outcome in children with growth hormone deficiency. Sci. Rep. **9**, 16181 (2019). 10.1038/s41598-019-52644-1 31700044 10.1038/s41598-019-52644-1 PMC6838176

[36] O. Abawi, D. Augustijn, S.E. Hoeks, Y.B. de Rijke, E.L.T. van den Akker, Impact of body mass index on growth hormone stimulation tests in children and adolescents: a systematic review and meta-analysis. Crit. Rev. Clin. Lab Sci. **58**(8), 576–595 (2021). 10.1080/10408363.2021.1956423 34431447 10.1080/10408363.2021.1956423

[37] S. Shalitin, G. Gat-Yablonski, Associations of obesity with linear growth and puberty. Horm. Res. Paediatr. **95**(2), 120–136 (2022). 10.1159/000516171 34130293 10.1159/000516171

[38] E. Kempf, M. Vogel, T. Vogel, J. Kratzsch, K. Landgraf, A. Kühnapfel, R. Gausche, D. Gräfe, E. Sergeyev, R. Pfäffle, W. Kiess, J. Stanik, A. Körner, Dynamic alterations in linear growth and endocrine parameters in children with obesity and height reference values. EclinicalMedicine **37**, 100977 (2021). 10.1016/j.eclinm.2021.100977 34386750 10.1016/j.eclinm.2021.100977 PMC8343253

[39] Z.U. Kareem, S.K. Panuganti, S: Bhatia, Case report: energy- and nutrient-dense formula for growth faltering: a report of two cases from India. Front. Nutr. **8**, 588177 (2021). 10.3389/fnut.2021.588177 33718416 10.3389/fnut.2021.588177 PMC7952321

[40] World Health Organization (WHO): Growth reference data for 5 to 19 years. Indicators. Bmi-for-age. https://www.who.int/tools/growth-reference-data-for-5to19-years/indicators/bmi-for-age (2024). Accessed 23 Jan 2024

[41] A.R. Kansra, S. Lakkunarajah, M.S. Jay, Childhood and adolescent obesity: a review. Front. Pediatr. **8**, 581461 (2021). 10.3389/fped.2020.581461 33511092 10.3389/fped.2020.581461 PMC7835259

[42] C. De Leonibus, S. De Marco, A. Stevens, P. Clayton, F. Chiarelli, A. Mohn, Growth hormone deficiency in prepubertal children: predictive markers of cardiovascular disease. Horm. Res. Paediatr. **85**(6), 363–371 (2016). 10.1159/000444143 26960169 10.1159/000444143

[43] S. Cianfarani, Safety of pediatric rhGH therapy: an overview and the need for long-term surveillance. Front. Endocrinol. **12**, 811846 (2021). 10.3389/fendo.2021.811846 10.3389/fendo.2021.811846 PMC8740026 35002983

